# Targeting cyclophilin-D by compound 19 protects neuronal cells from oxygen glucose deprivation/re-oxygenation

**DOI:** 10.18632/oncotarget.21655

**Published:** 2017-10-06

**Authors:** Jinyu Zheng, Enhui Cui, Haikou Yang, Mao Li, Jing Zhou, Ming Yan, Jian Sun, De-Rong Tang

**Affiliations:** ^1^ Department of Neurosurgery, The Affiliated Huai’an Hospital of Xuzhou Medical College, Huai’an, China; ^2^ Department of Anesthesiology, Huai’an Maternity and Child Healthcare Hospital, Yangzhou University Medical School, Huai’an, China; ^3^ Department of Thoracic Surgery, Huai’an First People's Hospital, Nanjing Medical University, Huai’an, China

**Keywords:** oxygen glucose deprivation/re-oxygenation, cyclophilin-D, compound 19, programmed necrosis, P53

## Abstract

Oxygen and glucose deprivation (OGD) with re-oxygenation (OGDR) is applied to neuronal cells to mimic ischemia-reperfusion injuries. Activation of cyclophilin D (Cyp-D)-dependent programmed necrosis pathway mediates OGDR-induced neuronal cell damages. Here, we tested the potential effect of Compound 19 (C19), a novel Cyp-D inhibitor, in this process. In both established neuronal cell lines (Neuro-2a and NB41A3 cells) and the primary murine CA1 hippocampal neurons, pretreatment with C19 largely attenuated OGDR-induced cell viability reduction and cell death. Significantly, C19 was ineffective in Cyp-D-silenced Neuro-2a cells. OGDR induced mitochondria-dependent programmed necrosis in neuronal cells. OGDR induced p53 translocation to mitochondria and association with Cyp-D, causing mitochondrial depolarization, cytochrome C release and reactive oxygen species production. Such effects were largely attenuated with pre-treatment of C19. Importantly, C19 was significantly more efficient than other known Cyp-D inhibitors in protecting neuronal cells from OGDR. These results suggest that targeting Cyp-D by C19 protects neuronal cells from OGDR.

## INTRODUCTION

Ischemia-reperfusion causes severe neuronal damages in the process of stroke and other neurological diseases [1, 2]. Oxygen and glucose deprivation (OGD) insult, followed by re-oxygenation (OGDR), is applied in cultured neuronal cells to mimic the pathological condition of ischemia-reperfusion injuries [3–6]. Recent studies have suggested that OGDR mainly induces necrosis, but not apoptosis, in neuronal cells [5, 7, 8].

Necrosis is traditionally known as a passive cell death behavior. Recent studies, however, have suggested that cell necrosis could also be an active course [7–10]. This active process, also known as “programmed necrosis”, is mitochondrial dependent [7–10]. p53 is required in mediating the programmed necrosis pathway [7–10]. A number of stimuli, including hypoxia, calcium overload, UV radiation, and oxidative stresses, were able to induce p53 translocation to mitochondria, and it forms a complex with cyclophilin D (Cyp-D) [7–10]. The complexation will dictate Cyp-D translocation to inner mitochondrial membrane [11–13], causing mitochondrial depolarization, cytochrome C (Cyto-C) release, as well as reactive oxygen species (ROS) production, and eventually cell necrosis [14–16]. Pharmacological inhibition or genetic silence of the Cyp-D-P53 complex could efficiently protect cells from the above stimuli [14–16]. Several known Cyp-D inhibitors, including sanglifehrin A (SfA) and cyclosporine A (CsA), were shown to recue cells from programmed necrosis [14–16].

A very recent study by Zhao *et al*., suggested that the programmed necrosis probably also mediated SH-SY5Y cell injuries by OGDR [3]. OGDR induced Cyp-D-P53 association in the mitochondria [3]. Disruption of this complex by Cyp-D inhibitor CsA, or by shRNA-mediated silencing Cyp-D/p53, significantly inhibited OGDR-induced SH-SY5Y cell programmed necrosis [3]. On the other hand, exogenous over-expression of Cyp-D exacerbated SH-SY5Y cell necrosis by OGDR [3]. Additionally, OGDR is also shown to induce Cyp-D-dependent programmed necrosis in myocardiocytes [7, 8].Thus, activation of mitochondrial programmed necrosis pathway mediates OGDR-induced cell damages [3, 7, 8].

Very recent studies have characterized a novel, highly specific and potent Cyp-D inhibitor, namely Compound 19 (C19) [17, 18]. C19's binding to Cyp-D has an extremely high affinity. This compound could block Cyp-D at nM-μM concentrations [18]. Here, we show that targeting Cyp-D by C19 efficiently protects neuronal cells from OGDR.

## RESULTS

### C19 protects neuronal cells from OGD/re-oxygenation

**Figure 1:**
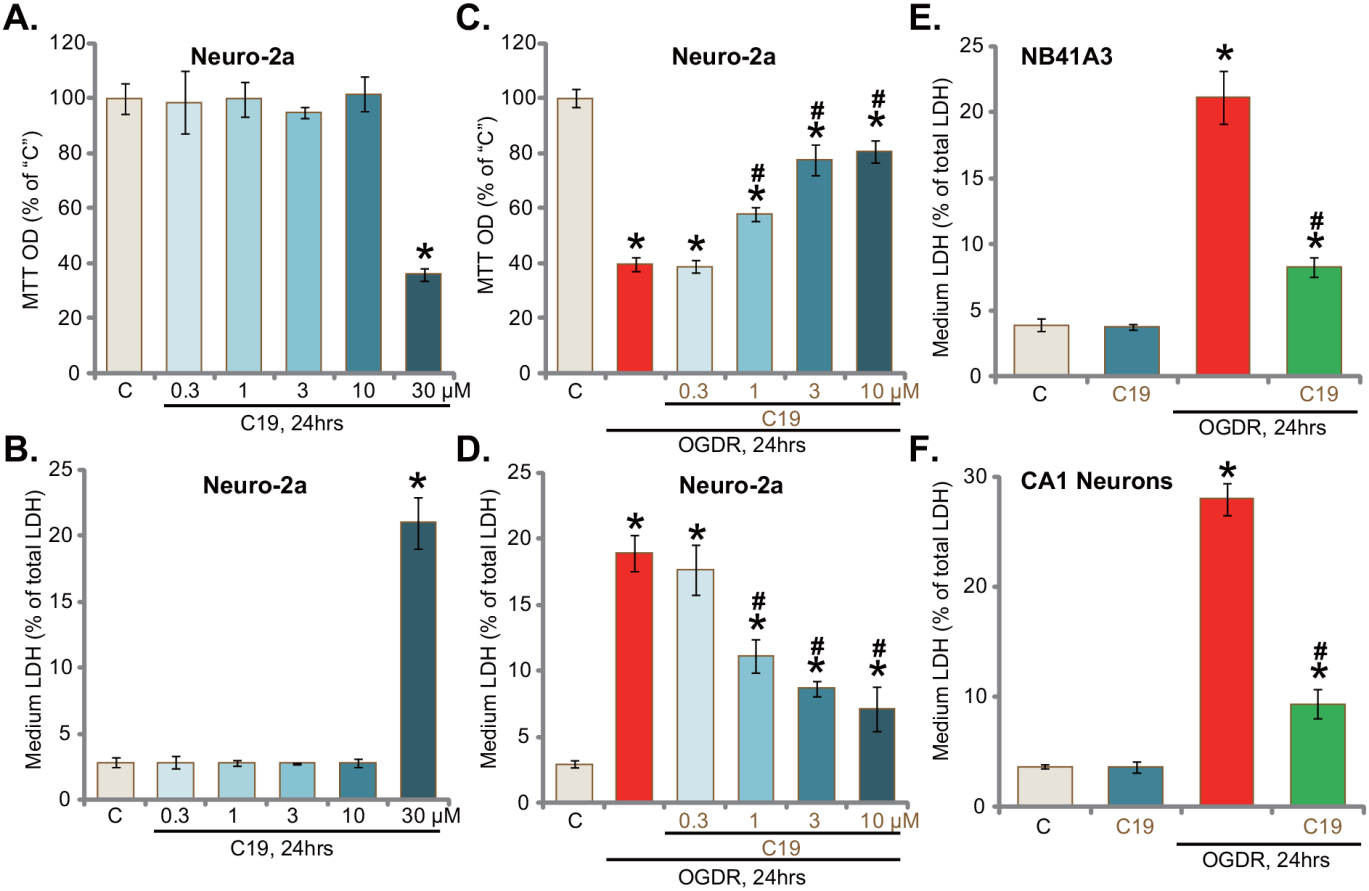
C19 protects neuronal cells from OGD/re-oxygenation Established murine neuronal cells (Neuro-2a and NB41A3 lines) or primary murine CA1 hippocampal neurons (“CA1 Neurons”) were pre-treated for 30 min with C19 at designated concentration, cells were then exposed to OGD for 6 hours, followed by 24 hours of re-oxygenation (“OGDR”); Cell survival was tested by MTT assay **(A** and **C)**; cell death was examined by LDH release assay **(B, D, E** and **F)**. “C” stands for “Mock” treatment (Same for all figures). “OGDR” stands for OGD/re-oxygenation (same for all figures). Bars indicate mean ± standard deviation (SD, n=5). ^*^ *p*<0.05 *vs.* “C” cells. ^#^ *p*<0.05 *vs.* “OGDR” only treatment. Each experiment was repeated three times with similar results obtained.

Neuro-2a is a well-established neuronal cell line [19]. Cultured Neuro-2a cells were treated with different concentrations of C19, the novel Cyp-D inhibitor [17, 18]. MTT assay was performed to test cell survival 24 hours after C19 treatment. Results in Figure 1A demonstrated that C19 was safe to Neuro-2a cells expect at a very high concentration (30 μM). LDH release assay results in Figure 1B showed that C19 was non-cytotoxic to Neuro-2a cells, till at the highest concentration (30 μM). Thus, C19 was tested at 0.3 to 10 μM for following experiments. As shown in Figure 1C, exposure of Neuro-2a cells with OGD (6 hours)/re-oxygenation (“OGDR”, 24 hours) caused over 50-60% reduction of cell viability (“MTT OD”). Pre-treatment for 30 min with C19 at 1-10 μM significantly attenuated OGDR's cytotoxicity (Figure 1C). Further, OGDR-induced Neuro-2a cell death, tested again by the LDH release in the condition medium, was also largely attenuated with C19 pretreatment (Figure 1D). C19 induced a concentration-dependent response in protecting Neuro-2a cells from OGDR (Figure 1C and 1D). Notably, C19, at a very low concentration (0.3 μM), was ineffective (Figure 1C and 1D). The potential effect of C19 on other neuronal cells was also analyzed. LDH release assay results confirmed that pre-treatment with C19 (10 μM, 30 min) largely attenuated OGDR-induced death (LDH release) of established NB41A3 neuronal cells (Figure 1E) and primary murine CA1 hippocampal neurons (Figure 1F). Treatment with C19 alone failed to induce LDH release of above neuronal cells (Figure 1E and 1F). Together, these results suggest that C19 protects neuronal cells from OGD/re-oxygenation.

### Cyp-D is the primary target protein of C19 in neuronal cells

**Figure 2:**
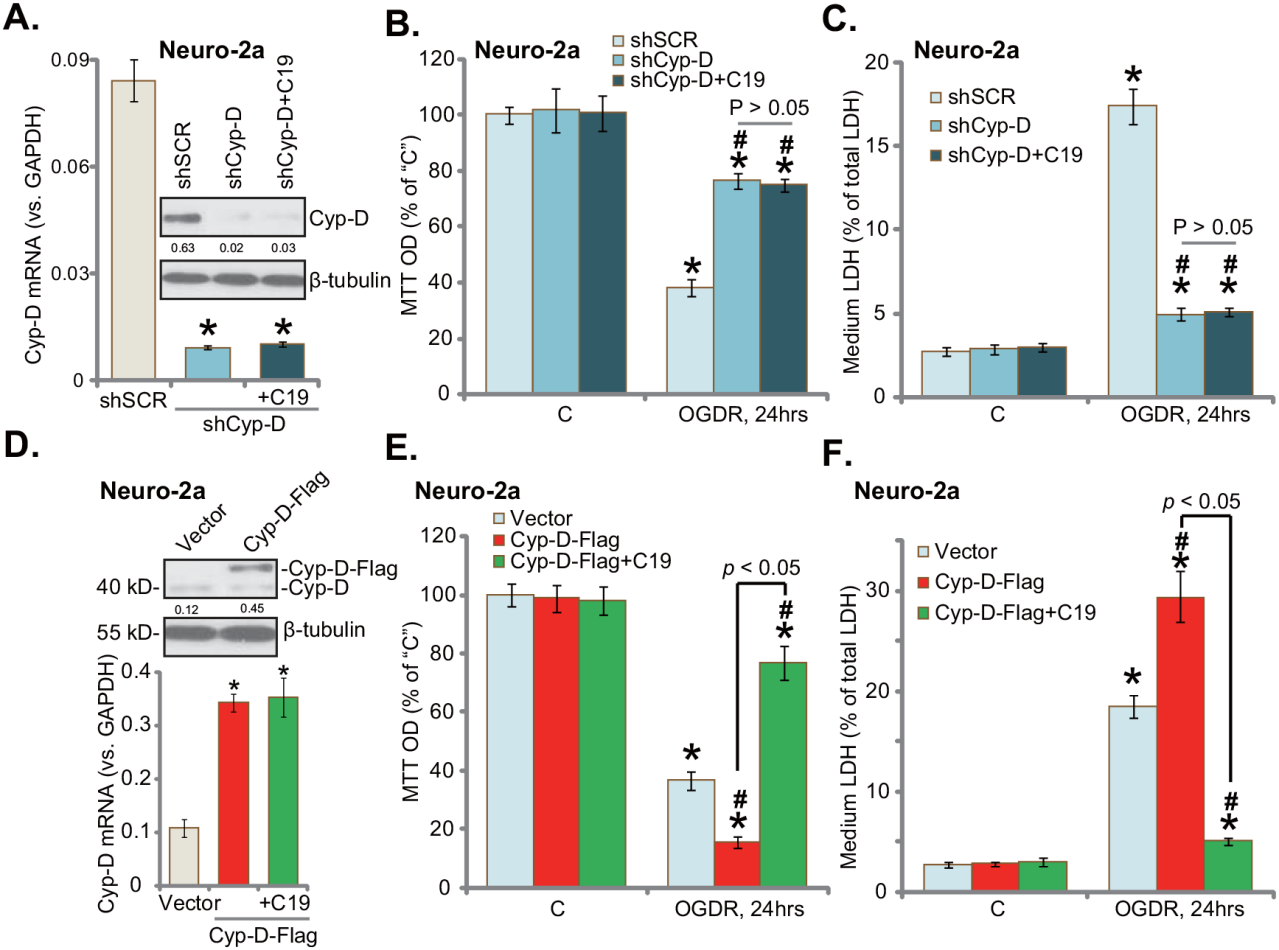
Cyp-D is the primary target protein of C19 in neuronal cells The puromycin-selected stable Neuro-2a cells, expressing Cyp-D shRNA (“shCyp-D”) or Cyp-D-cDNA vector (“Cyp-D-Flag”), were pre-treated with/out C19 (“+C19”, 10 μM, 30 min), cells were then exposed to OGD for 6 hours, followed by 24 hours of re-oxygenation (“OGDR”); expressions of *Cyp-D mRNA* and protein were shown **(A** and **D)**; cell survival was tested by MTT assay **(B** and **E)**; cell death was examined by LDH release assay **(C** and **F)**. Cyp-D protein expression was quantified and normalized to the loading control β-tubulin (A and D). “shSCR” stands for scramble control shRNA (A-C); “vector” stands for empty vector control cells (D-F). Bars indicate mean ± standard deviation (SD, n=5). ^*^ *p*<0.05 *vs.* “C” cells. ^#^ *p*<0.05 *vs.* “OGDR” of “shSCR” cells (A-C). ^#^ *p*<0.05 *vs*. “OGDR” of “Vector” cells (D-F). Each experiment was repeated three times with similar results obtained.

If Cyp-D is the primary target of C19, it should be ineffective in the Cyp-D-silenced cells. To test this hypothesis, shRNA strategy was applied to knockdown Cyp-D in Neuro-2a cells. The Cyp-D-shRNA lentiviral particles were added to cultured Neuro-2a cells, and puromycin was then added to establish the stable cells. Results from both the quantitative real-time PCR assay (“qRT-PCR” assay) and Western blotting assay confirmed dramatic Cyp-D knockdown (over 90%) in the stable Neuro-2a cells with the targeted shRNA (Figure 2A). Adding C19 failed to change Cyp-D protein/mRNA expression (Figure 2A). As demonstrated, stable Neuro-2a cells with Cyp-D shRNA were largely protected from OGDR (Figure 2B and 2C). Cyp-D-induced viability reduction (MTT OD decrease, Figure 2B) and cell death (LDH release, Figure 2C) were largely attenuated in Cyp-D-silenced Neuro-2a cells. These results support that Cyp-D is required for OGDR-induced cytotoxicity in neuronal cells. Remarkably, C19 was unable to further protect Cyp-D-silenced Neuro-2a cells from OGDR (Figure 2B and 2C). These results imply that Cyp-D should be the primary target protein of C19 in Neuro-2a cells.

Based on the results above, we proposed that Cyp-D over-expression should favor ODG-induced neuronal cell death. Therefore, a Cyp-D expression vector (see Method) was introduced to Neuro-2a cells. Puromycin was added again to select stable cells. Western blotting assay testing the stable cells confirmed expression of exogenous Cyp-D (tagged with Flag) (Figure 2D, upper panel). Cyp-D over-expression was also confirmed by the qRT-PCR assay (Figure 2D, lower panel). As displayed, Cyp-D over-expression indeed facilitated OGDR-induced neuronal cell viability reduction (Figure 2E) and cell death (Figure 2F). Significantly, pre-treatment with C19 (10 μM, 30 min) was able to largely attenuated OGDR-induced cytotoxicity in Cyp-D-over-expressed Neuro-2a cells (Figure 2E and 2F). The Cyp-D shRNA and over-expression experiments were also repeated in NB41A3 cells, and similar results were obtained (Data not shown). Notably, Cyp-D knockdown or over-expression alone failed to change Neuro-2a cell survival and death (Figure 2B, 2C, 2E and 2F). Collectively, these results suggest that Cyp-D is the primary target protein of C19 in neuronal cells.

### OGD/re-oxygenation fails to induce apoptosis in neuronal cells

**Figure 3:**
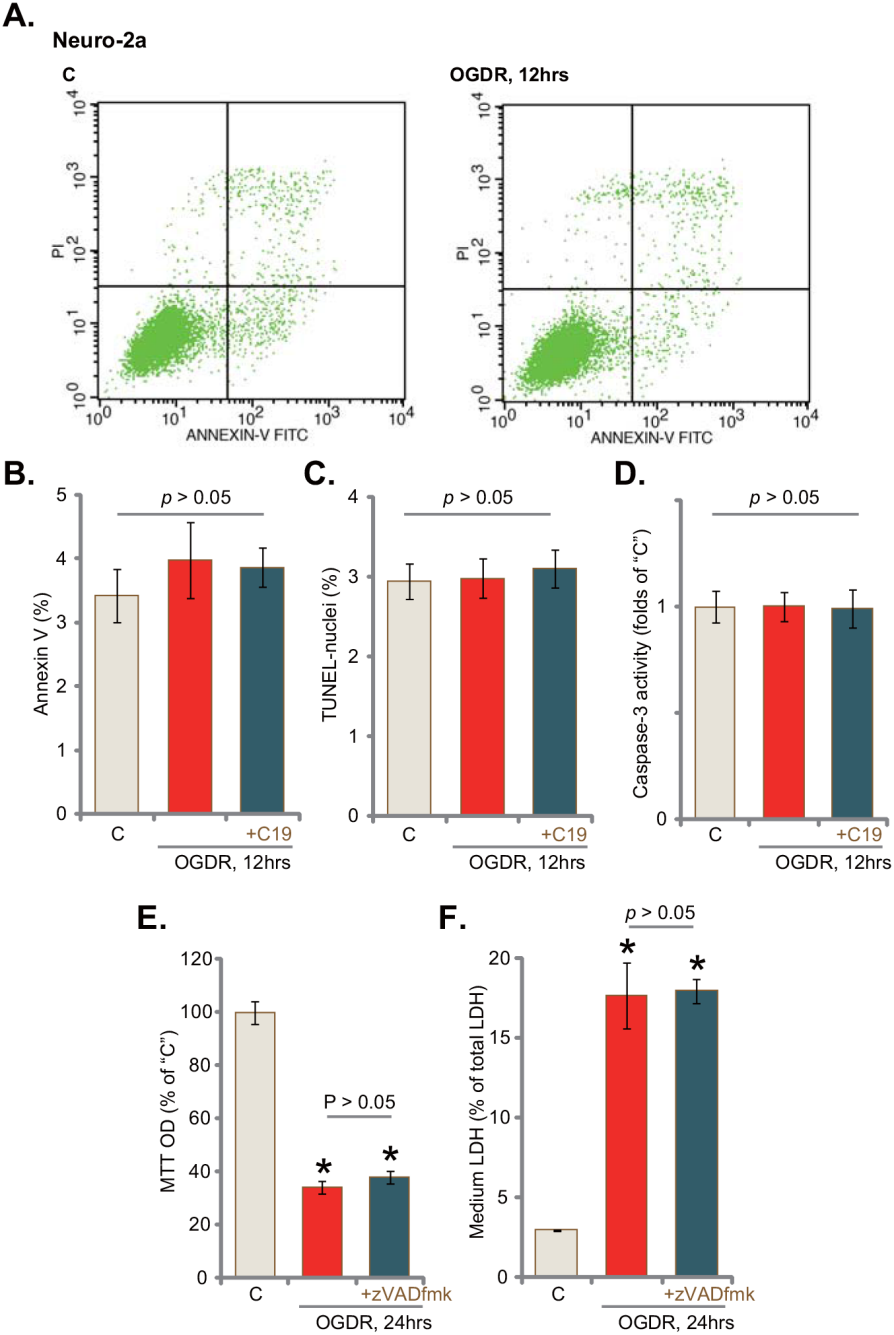
OGD/re-oxygenation fails to induce apoptosis in neuronal cells Neuro-2a cells were pre-treated with C19 (“+C19”, 10 μM, 30 min) or the pan caspase inhibitor zVADfmk (50 μM, 30 min), cells were then exposed to OGD for 6 hours, followed by re-oxygenation for applied time (“OGDR”); cell apoptosis was tested by the assays mentioned in the text **(A-D)**; cell viability (MTT assay, **(E)** and cell death (LDH assay, **(F)** were also tested. Bars indicate mean ± standard deviation (SD, n=5). ^*^ *p*<0.05 *vs*. “C” cells (E and F). Each experiment was repeated three times with similar results obtained.

Apoptosis induction shall be a major way of cell death. We therefore tested apoptosis level in OGDR-treated Neuro-2a cells. Different apoptosis assays were performed, including the Annexin V FACS assay, TUNEL staining assay and caspase-3 activity assay. Intriguingly, the results of these apoptosis assays failed to detect significant apoptosis activation in OGDR-treated Neuro-2a cells (Figure 3A–3D). Following OGDR treatment, Annexin V percentage (Figure 3A–3B), TUNLE-nuclei ratio (Figure 3C) and the caspae-3 activity (Figure 3D) were not significantly changed in Neuro-2a cells. Pre-treatment with C19 (10 μM, 30 min) also failed to change cell apoptosis level (Figure 3A–3D). Further, z-VAD-fmk, a pan caspase inhibitor, was unable to rescue Neuro-2a cells from OGDR (Figure 3E and 3F). Note that the above non-apoptosis results were also observed in OGDR-treated NB41A3 cells and CA1 neurons (Data not shown). Therefore, there should be another form of non-apoptotic cell death by OGDR. These results suggest that OGDR mainly induces non-apoptotic death in neuronal cells.

### C19 inhibits OGD/re-oxygenation-induced programmed necrosis in neuronal cells

**Figure 4:**
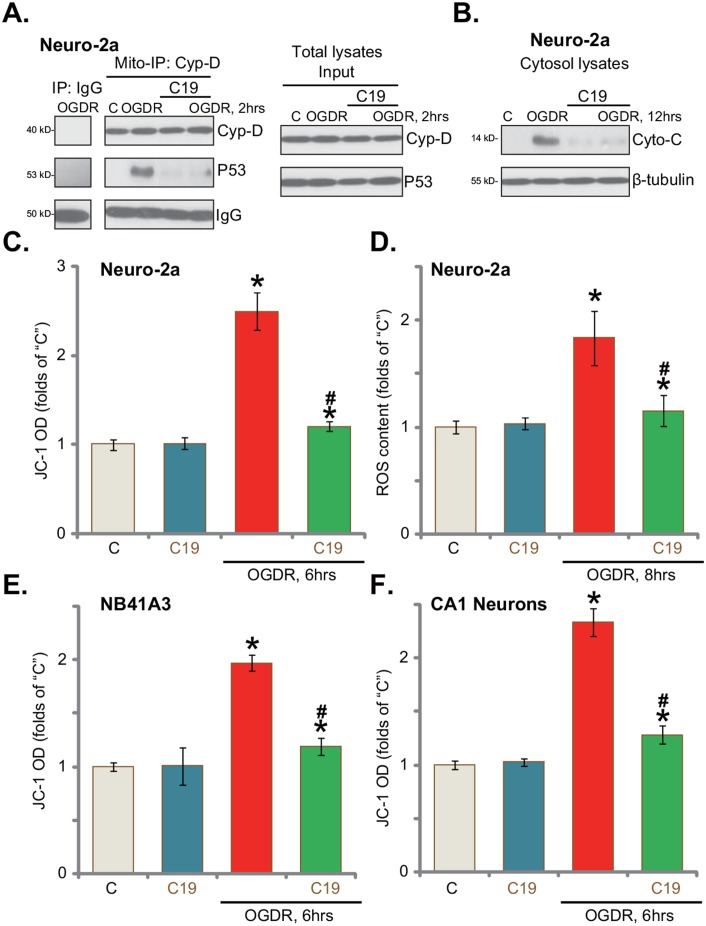
C19 inhibits OGD/re-oxygenation-induced programmed necrosis in neuronal cells Neuro-2a cells **(A-D)**, NB41A3 cells **(E)** or primary murine CA1 hippocampal neurons **(F)** were pre-treated with C19 (10 μM, 30 min), cells were then exposed to OGDR for 6 hours, followed by re-oxygenation for applied time (“OGDR”); Cyp-D-p53 mitochondrial association (“Mito-IP” assay, in mitochondrial lysates) and expressions (“Input”, in total lysates) were tested (A); mitochondrial depolarization (JC-1 green fluorescence intensity OD increase, C, E and F) and cytochrome c (“Cyto-C”) release (B) were also tested. Relative ROS intensity was tested by the DCFH-DA dye assay (D). Bars indicate mean ± standard deviation (SD, n=5). ^*^ *p*<0.05 *vs.* “C” cells. ^#^ *p*<0.05 *vs.* “OGDR” only treatment. Each experiment was repeated three times with similar results obtained.

The above-mentioned results proposed that there should be a non-apoptotic form of cell death following OGDR treatment. Recent studies have proposed a mitochondria-dependent active necrosis pathway, also named as programmed necrosis, that can be induced under many stress conditions. Different stresses, including hypoxia, calcium overload, and oxidative stress, could lead to p53 mitochondrial translocation, which associates with the local protein Cyp-D [3, 20–23]. Thereafter, Cyp-D-p53 association triggers mitochondrial depolarization, cytochrome C release and cell necrosis (but not apoptosis) [3, 20–23]. Here, the mitochondria immunoprecipitation (“Mito-IP”) assay results confirmed the association between Cyp-D and p53 in the mitochondria of OGDR-treated Neuro-2a cells (Figure 4A). Such association was not observed in the control Neuro-2a cells (Figure 4A). Notably, Cyp-D-p53 association was followed by cytochrome C release to cytosol (Figure 4B) and mitochondrial depolarization (JC-1 green fluorescence intensity increase, Figure 4C). Remarkably, such effects were largely inhibited by pre-treatment with C19 (Figure 4A–4C). Based on the results above, we would propose that C19, as a Cyp-D inhibitor, likely blocked programmed necrosis pathway, and protected neuronal cells from OGDR (Figure 4A–4C). Importantly, LDH release (See Figures 1–3) in OGDR-treated cells is also a characteristic marker of cell necrosis, but not apoptosis.

Recent studies have suggested that mitochondrial programmed necrosis pathway activation is always accompanied with reactive oxygen species (ROS) production and oxidative stress [3, 20–23]. In fact, ROS level was significantly increased in OGDR-treated Neuro-2a cells (Figure 4D), which was again largely inhibited by C19 pre-treatment (Figure 4D). In the NB41A3 cells (Figure 4E) and primary murine CA1 hippocampal neurons (Figure 4F), C19 pre-treatment also largely inhibited mitochondrial depolarization (JC-1 assay). Thus, C19 apparently inhibits OGDR-induced activation of programmed necrosis pathway.

### C19 is more efficient than other known Cyp-D inhibitors in protecting neuronal cells from OGD/re-oxygenation

**Figure 5:**
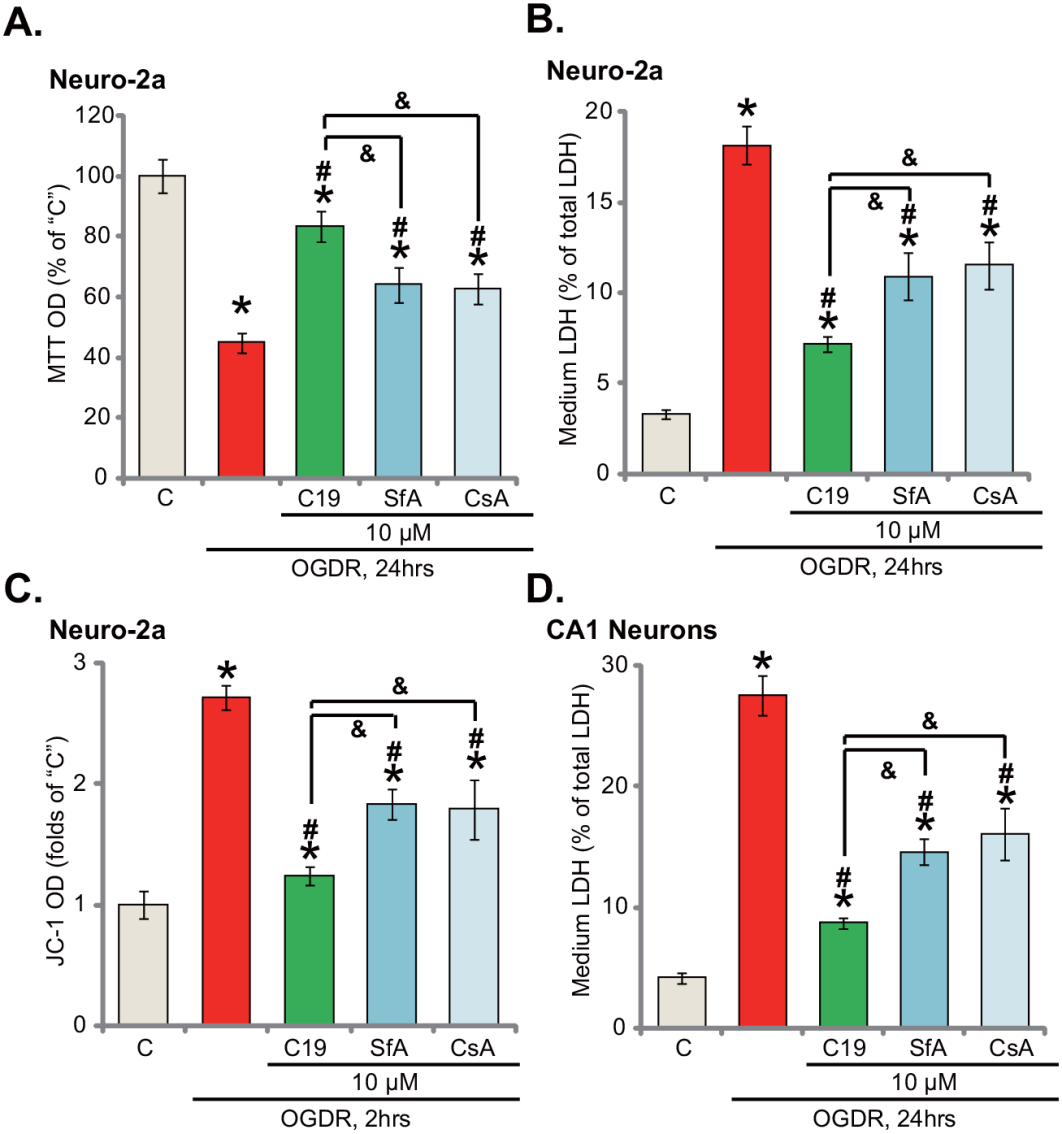
C19 is more efficient than other known Cyp-D inhibitors in protecting neuronal cells from OGD/re-oxygenation Neuro-2a cells **(A-C)** or primary murine CA1 hippocampal neurons **(D)** were pre-treated for 30 min with 10 μM of C19, cyclosporin A (CsA) or sanglifehrin A (SfA), cells were then exposed to OGD for 6 hours, followed by re-oxygenation for applied time (“OGDR”); cell survival (MTT assay, A), cell death (LDH release assay, B and D) and mitochondrial depolarization (JC-1 green fluorescence OD increase, C) were tested. Bars indicate mean ± standard deviation (SD, n=5). ^*^ *p*<0.05 *vs.* “C” cells. ^#^ *p*<0.05 *vs.* “OGDR” only treatment. & *p*<0.05. Each experiment was repeated three times with similar results obtained.

We also compared the activity of C19 with other known Cyp-D inhibitors, including cyclosporin A (CsA) [24] and sanglifehrin A (SfA) [25]. Results demonstrated that pre-treatment for 30 min with CsA (10 μM) or CsA (10 μM) also attenuated OGDR-induced Neuro-2a cell viability reduction (Figure 5A) and cell death (Figure 5B). Yet, the same concentration of C19 (10 μM) showed highest efficiency in protecting Neuro-2a cells (Figure 5A and 5B). Thus, C19 is apparently more potent in attenuating OGDR damages than the known Cyp-D inhibitors (CsA and SfA). Further studies demonstrate that C19-induced inhibition on mitochondrial depolarization (JC-1 OD increase) in OGDR-treated cells was also more potent than CsA or SfA (Figure 5C). Therefore, targeting Cyp-D by C19 is quite efficient in shutting down the programmed necrosis pathway. Notably, as shown in Figure 5D, C19-mediatd cytoprotection against OGDR in CA1 hippocampal neurons was also most efficient among all tested Cyp-D inhibitors.

## DISCUSSION AND CONCLUSION

p53 is critical in mediating cell apoptosis [26–30]. Intriguingly, very recent studies have demonstrated that p53 is also actively involved in cell necrosis, a process that is known as “programmed necrosis” [23, 31, 32]. Following different stimuli, *i.e.* hypoxia, calcium overload, UV radiation, and oxidative stresses, p53 translocates to mitochondria, and forms a complex with Cyp-D. This Cyp-D-p53 association in the mitochondria is essential for the mitochondrial depolarization, following mitochondrial transition pore opening (mPTP) opening [9], and more importantly, subsequent cell necrosis (but not apoptosis) [23, 31, 32]. Genetic or pharmacological inhibition of Cyp-D-p53 complex could then efficiently protect cells from above stresses [23, 31, 32].

Very recent studies have developed a novel and specific Cyp-D inhibitor, namely C19 [18]. This novel Cyp-D inhibitor has shown to inhibit toxin-induced mitochondrial depolarization and necrotic cell death [18]. Our results here demonstrate that C19 efficiently protected neuronal cells from OGDR. Targeting Cyp-D by C19 almost completely blocked OGDR-induced mitochondrial Cyp-D-p53 binding, mitochondrial depolarization as well as cytochrome C release and ROS production. Consequently, OGDR-induced neuronal cell necrosis was also alleviated with C19 pretreatment. Therefore, C19-mediated neuronal cytoprotection is possibly due to blockage of Cyp-D mitochondrial necrosis pathway.

As compared to other known Cyp-D inhibitors (*i.e*. CsA [33] and SfA [25]), C19 displayed a better K_d_ (nM-μM concentrations), a fine thermodynamic profile, along with improved pharmacokinetic/pharmacodynamic property [18]. More importantly, this novel Cyp-D inhibitor showed extreme high selectivity to Cyp-D [18]. In the current study, we show that C19 was significantly more efficient than other known Cyp-D inhibitors (CsA and SfA) in protecting neuronal cells from OGDR. More importantly, we propose that Cyp-D is the primary target protein of C19. Cyp-D knockdown by targeted-shRNA similarly protected Neuro-2a cells from OGDR. Significantly, C19 was ineffective in Cyp-D-silenced Neuro-2a cells.

The neuronal death following ischemic stroke cannot be well controlled due to the lack of efficient therapeutic or neuroprotective methods. Thus, there is an urgent need to develop more effective treatments for stroke [34–36]. The results of this current study demonstrated that targeting Cyp-D by C19 efficiently protected neuronal cells from OGDR. Thus, C19 could possibly be further tested as a promising anti-ischemic stroke agent to protect neurons.

## MATERIALS AND METHODS

### Chemical and reagents

Compound 19 (“C19”) was provided by Dr. Song at Soochow University [17]. The known Cyp-D inhibitors, including sanglifehrin A (SfA) and cyclosporine A (CsA) [37], were purchased from Sigma-Aldrich (Shanghai, China). Puromycin was also obtained from Sigma-Aldrich. The murine Cyp-D shRNA lentiviral particles and the scramble control lentiviral particles were obtained from Santa Cruz Biotech (Santa Cruz, CA). The cell culture reagents were provided by Gibco (Nanjing, Jiangsu, China). The antibodies for p53, Cyp-D, cytochrome C (Cyto-C) and β-tubulin were purchased from Santa Cruz Biotechnology (Santa Cruz, CA).

### Culture of established neuronal cell lines

The neuroblastoma cell lines, Neuro-2a and NB41A3, were purchased from the Cell Bank of Biological Institute of Chinese Academy of Science (Shanghai, China). Cells were cultured in DMEM with 10% fetal bovine serum (FBS), plus penicillin/streptomycin (1:100, Sigma), and 4 mM L-glutamine and 0.25% HEPES (Sigma).

### Primary culture of CA1 neurons

Primary murine neurons were prepared from CA1 hippocampus of E14-E16 embryos. CA1 neurons (200,000 cells/cm^2^) were plated in serum-free neuron basal medium with 2% B27 supplement and 2 mM glutamine. On day 10 of culture, the majority (> 95%) of cells were neurons.

### Cell viability assay

The viability of neuronal cells was examined by the routine MTT assay (Sigma) according to the recommended protocol. MTT OD at 490 nm was recorded.

### LDH assay

The release of lactate dehydrogenase (LDH) is a characteristic marker of cell necrosis. The measurement of released LDH to the conditional medium was examined via using a commercial available two-step LDH detection kit (Promega, Shanghai, China). LDH content in the conditional medium was normalized to the total LDH.

### OGD/re-oxygenation

The detailed procedure of OGD/re-oxygenation (“OGDR”) was described previously [3, 5, 7, 8, 38, 39]. In brief, the neuronal cells were placed in an airtight chamber and equilibrated for 10 min with a continuous flux of gas (95% N_2_/5% CO_2_). The chamber was sealed and placed in an incubator for 6 hours OGD. Cells were then re-oxygenated for indicated time. Control cell cultures that were not deprived of oxygen and glucose, were placed in norm-oxygenated DMEM containing glucose.

### Western blotting assay

The RIPA lysis buffer (purchased from Biyuntian, Wuxi, China) was applied to obtain the cellular lysate samples, which was then normalized, and boiled in SDS sample buffer. To avoid protein degradation, a mixture of proteinase inhibitors were added. The protein lysate samples (30 μg per treatment) were then separated by the 10-12% SDS-PAGE gels, and were transferred to the polyvinylidene difluoride (PVDF) membrane. The blot was then blocked with 10% milk in PBST, and labeled with indicated primary and secondary antibodies. The super-signal West Pico Enhanced Chemiluminescent (ECL) reagents (Amersham, Shanghai, China) were added to visualize the targeted protein band under X-ray film development [40–42]. Each band was quantified via the ImageJ software, and was normalized to the corresponding loading control.

### Caspase-3 activity assay

The cytosol proteins of approximately one million cells per treatment were extracted in cell lysis buffer as described [22]. Twenty μg of cytosolic extracts were added to caspase assay buffer [22] with the caspase-3 substrate [22]. After 2 hours of incubation, the release of 7-amido-4-(trifluoromethyl) coumarin (AFC) was quantified, via a Fluoroskan system set [22]. The AFC OD value (at 405 nm) of treatment group was always normalized to that of control group.

### Annexin V FACS assay of cell apoptosis

Following the indicated treatment, neuronal cells (1×10^5^ cells per sample) were washed with PBS, and incubated with Annexin V-FITC (5 μL/mL medium, Invitrogen) and propidium iodide (PI, Invitrogen). Cells were then subjected to fluorescence-activated cell sorting (FACS) of Annexin V/PI, using a Becton-Dickinson FACScan (Shanghai, China).

### TUNEL assay

The TUNEL *In Situ* Cell Apoptosis Detection Kit (Roche, Shanghai, China) was utilized to quantify apoptosis in neuronal cells. TUNEL-positive stained nuclei were visualized under a fluorescence microscopy (Leica DM2500). TUNEL ratio (*vs.* total number of cell nuclei, Hoechst-stained) was calculated, from at least 200 cells of six random views for each treatment.

### Mitochondrial immunoprecipitation (Mito-IP)

For each treatment, 10 million neuronal cells were harvested and homogenized using the buffer A (250 mM sucrose, 20 mM HEPES, 10 mM KCl, 1.5 mM MgCl_2_, 1 mM EDTA, 1 mM EGTA, and 1 mM dithiothreitol). After centrifugation, the supernatants were collected as the cytosolic fractions. The pellets were then re-suspended in buffer B (1 mL buffer A containing 10 μL NP-40). After centrifugation, the supernatants were collected as the “mitochondrial fractions”. The pre-cleared mitochondrial lysates (500 μg per treatment) were incubated with anti-Cyp-D antibody ([8, 9]). The mitochondrial immune complexes were then captured with protein A/G-Sepharose beads (Sigma, Shanghai, China). Cyp-D-p53 association was then tested by Western blotting assay.

### Mitochondrial depolarization assay

JC-1 fluorescent dye is a reliable indicator of the mitochondrial membrane potential changes in intact cells [43]. During mitochondrial depolarization, the red JC1 aggregates form green monomers due to a change in ΔΨ [44]. The detailed JC-1 protocol testing mitochondrial depolarization was described in previous studies [20, 22, 45–47]. Briefly, neuronal cells with the indicated treatment were washed with warm PBS, and then stained with JC-1 (5 μg/mL, Invitrogen, Shanghai, China) for 10 min. JC-1 green intensity, indicating mitochondrial depolarization, was examined immediately on a fluorescence spectrofluorometer at 550 nm (Titertek Fluoroscan, Germany).

### Reactive oxygen species (ROS) detection

The 2′,7′-dichlorofluorescein diacetate (DCFH-DA) dye assay was performed to test cellular ROS intensity [48–50]. DCFH-DA freely penetrates cells, and is then hydrolysed by intracellular esterases to DCFH. Following the applied treatment, cells were stained with DCFH-DA (100 μM, Invitrogen) for 60 min. Cells were then washed with PBS for three times. DCF fluorescence signal was detected by the fluorescence microplate reader (Titertek Fluoroscan, Germany). ROS intensity in the treatment cells was always normalized to that of control cells.

### qRT-PCR

Following the applied treatment, total cellular RNA was extracted via using the TRIzol reagents (Promega, Shanghai, China) [51]. The SYBR Green PCR kit (Applied Biosystems, Suzhou, China) was utilized for reverse transcription. The ABI Prism 7600 Fast Real-Time PCR system was utilized to perform the quantitative real time-PCR (qRT-PCR) assay. For each assay, melt curve analysis was performed to calculate product melting temperature. *Glyceraldehyde-3-phosphatedehydrogenase* (*GAPDH*) mRNA was chosen as the reference gene, and the 2^−ΔΔ*C*t^ method was applied to quantify Cyp-D mRNA expression change. The murine *Cyp-D mRNA* primers were 5’-AGGTGGCGAAAGTATTTATG-3’ and 5’-GGAGTCGGAACTGTTGTGAT-3’ [52].

### Cyp-D knockdown by targeted-shRNA

Neuronal cells were cultured at 50% confluence in low-serum (2%) medium without antibiotics. The murine Cyp-D shRNA lentiviral particles (10 μL/mL medium) or the scramble control shRNA lentiviral particles (10 μL/mL medium) was added to the cultured neuronal cells for 24 hours. Afterwards, cells were cultured in puromycin (3.0 μg/mL)-containing complete medium for another 6-8 days, and the stable cells were established. Cyp-D expression in the stable cells was tested by Western blot assay and qRT-PCR assay.

### Cyp-D over-expression

The Cyp-D expression vector (with both GFP and puromycin-resistance gene) and the empty vector (“pSuper-puro-GFP”) were provided by Dr. Bi at Nanjing Medical University [53]. Lipofectamine 2000 reagent (Invitrogen, Suzhou, China) was applied to transfect Cyp-D construct (or the empty vector) to the neuronal cells. Thirty-six hours after transfection, cells were cultured in puromycin (3.0 μg/mL)-containing complete medium for another 6-8 days, and the stable cells were established. Cyp-D expression in the stable cells was tested by Western blot assay and qRT-PCR assay.

### Statistics

The data were presented as mean ± standard deviation (SD). Statistical differences were analyzed by one-way ANOVA with post hoc Bonferroni test (SPSS version 18.0). Values of *p* < 0.05 were considered statistically significant.
